# Knowledge, Attitude, and Practices of Moroccan Retail Pharmacists towards Veterinary Medicines

**DOI:** 10.1155/2022/9973945

**Published:** 2022-01-04

**Authors:** Zeinab Snoussi, Samir Ahid

**Affiliations:** ^1^Pharmacoepidemiology and Pharmacoeconomics Research Team, Laboratory of Pharmacology & Toxicology, Faculty of Medicine and Pharmacy, Mohammed V University, Rabat, Morocco; ^2^Methodological Support Unit, Faculty of Pharmacy, Mohammed VI University of Health Science, Casablanca, Morocco

## Abstract

This is the first study conducted in Morocco to assess knowledge, attitude, and practices of retail pharmacists regarding veterinary medicines. It is a cross-sectional study. Two types of multiple-choice questionnaires were distributed to pharmacists depending on whether or not they dispense veterinary medicines. A total of 143 pharmacists were involved in this study. The percentage of retail pharmacists who dispensed veterinary medicines was estimated at 23.1%. Less than half of respondents were highly satisfied regarding their knowledge of veterinary pharmacy. Besides, 39.4% of retail pharmacists were at ease giving advice in general while dispensing veterinary medicines, and 73% were knowledgeable on parasiticides. Approximately, 94% of retail pharmacists expressed their need to improve their knowledge of veterinary pharmacy. Our study also revealed that 48.5% of pharmacists dispensed veterinary medicine daily. This study demonstrated that involvement of retail pharmacists in dispensing veterinary medicines was poor. The need for training programs on veterinary pharmacy expressed by Moroccan retail pharmacists was high.

## 1. Introduction

The World Organisation for Animal Health (OIE) defines veterinary products as tools for preventing and controlling animal diseases. Such products comprise vaccines, veterinary medicines, and diagnostic kits [1].

In Morocco, veterinary pharmacy is governed by law number 21-80, dated December 26^th^, 1980, related to veterinary medicine, surgery, and pharmacy and by its implementing decree n° 2-82-541 of March 15^th^, 1983 [2]. Under Moroccan law number 21-80, veterinary medicines include products or preparations, known for having curative or preventive properties regarding animal diseases and for medical diagnosis or restoring, correcting, or modifying its organic functions. The law includes any mixture of medicines and food prepared in advance and presented for administration to animals without transformation for therapeutic, preventive, or curative purposes [3].

In addition to medicines, veterinary pharmacy includes biocides, nutritional supplements, and premixes of authorized additives [4]. Law n° 17-04 on the Medicinal and Pharmaceutical Code regulates the dispensing of veterinary medicinal products among pharmacists [5].

The Division of Pharmacy and Veterinary Inputs (DPIV) is the institution in charge of the registration of veterinary medicines and the inspection of pharmaceutical companies and establishments for the production and distribution of veterinary medicines and inputs. This institution is responsible for veterinary pharmacovigilance. Established in 1950, this institution is under the authority of the National Office of Food Safety (ONSSA) [4]. The Ministry of Health is also involved in the veterinary pharmacy sector through the Directory of Medicines and Pharmacy (DMP). This institution is responsible, in collaboration with the DPIV, for analysis and tests required for veterinary medicines' control and for the marketing authorization process [6].

Until January 2020, 32 pharmaceuticals companies have been authorized by the DPIV and approximately 1993 veterinary medicines have obtained marketing authorization according to law n° 21-80. These companies were mostly located in the region of Casablanca and Rabat [7]. Veterinary medicines in Morocco are mainly composed of antibiotics, parasiticides, anti-inflammatories, and vaccines [8].

In Morocco, there are neither previous studies nor data about dispensing of veterinary medicines by retail pharmacists. We sought to provide a description of pharmacists' knowledge, attitudes, and practices of dispensing veterinary medicines. Concerning pharmacists who did not dispense veterinary medicines, we aim to understand the reasons for not dispensing these medicines.

## 2. Methods

This is a cross-sectional study on veterinary medicines in Moroccan retail pharmacies.

### 2.1. Study Groups

We included retail pharmacists working in different regions of Morocco. Pharmacists who dispensed veterinary medicines are defined as Group 1 and pharmacists with no dispensing experience as Group 2.

### 2.2. Data Collection and Processing

Data were collected using a multiple-choice questionnaire distributed to pharmacists at scientific events, in retail pharmacies, and by email using Google Forms. The questionnaire was filled out by pharmacists themselves. The pilot study which assessed the suitability of the questionnaire involved six pharmacists in Rabat using an evaluation grid. The final multiple-choice questionnaire was split into two types depending on whether pharmacist dispensed or not veterinary medicines (Group 1 and Group 2). The multiple-choice questionnaire Q1 for Group 1 was composed of 33 questions divided into seven sections: personal and professional data, information on veterinary medicines, community pharmacy practice, dispensary advice, economic aspects of veterinary medicines, veterinarian and pharmacist relationships, and training in veterinary pharmacy. The second questionnaire Q2 for Group 2 was shorter and included only three sections: personal and professional data, training on veterinary pharmacy, and reasons for not dispensing veterinary medicines.

Retail pharmacies were selected on a convenience sampling basis. Also, an electronic copy of the questionnaire was developed using Google Forms and mailed to pharmacists.

Pharmacists were reminded to complete the questionnaire left in the pharmacies by regular telephone calls. They were also called to check whether they completed the questionnaire. Uncompleted questionnaires were excluded from the study.

### 2.3. Statistical Analysis

The data were entered in Excel according to a predefined coding. Statistical analysis was performed using the SPSS 13.0 software. For quantitative data, the mean and standard deviation were calculated, and the frequency was calculated for qualitative data. The threshold of significance *p* is less than 0.05 (the confidence interval is 95%).

### 2.4. Ethical Approval

Ethical approval was obtained from the Biomedical Research Ethics Committee of the Faculty of Medicine and Pharmacy of Rabat before starting the study. Consent was requested from pharmacists before filling out the questionnaire.

## 3. Results

The questionnaires were distributed by three investigators who explained the purpose and modalities of this study. Pharmacies included in the study were located in 9 of 12 Moroccan regions. Distributing questionnaires took approximately four months. Among 157 questionnaires, 104 were distributed at scientific events, 42 in retail pharmacies, and 11 using Google Forms.

The percentage of women who responded was estimated at 37.1%. Besides, 83.2% of pharmacists worked in urban areas and 46.9% had studied in Eastern Europe (Table 1).

The percentage of pharmacists dispensing veterinary medicines (Group 1) was estimated at 23.1% (33). Less than half of them were highly satisfied regarding their knowledge of veterinary pharmacy, and 9% were dissatisfied.

Sixty-nine percent did not have courses in veterinary pharmacy during their undergraduate studies, and 59.4% did not receive tuition in veterinary pharmacy during postgraduate studies. However, 93.5% expressed their need to improve their knowledge of veterinary pharmacy.

The veterinary medicine represented less than one-third of overall medicine stock for 71.9% of pharmacies and more than one-third for 12.5% of pharmacies.

All pharmacies stocked parasiticides for animal treatment, while 94% of pharmacies stocked veterinary medicines to treat gastrointestinal conditions in animals.

Overall, 39.4% of pharmacists felt comfortable in dispensing advice in general on these medicines, but only 3% were not. Of the 33 pharmacists, 48.5% dispensed veterinary medicine daily and 39.4% weekly. Regarding forms of veterinary medicine dispensing in pharmacies, 57.1% of pharmacists declared they rarely received a veterinary prescription (Table 2). When they were asked to dispense prescribed veterinary medicines, 51.5% of pharmacists felt comfortable. Often, pharmacists sold veterinary medicines at the request of the animal owner (60.6%).

Parasiticides were the best-selling therapeutic class (69.6%) followed by antibiotics (15.1%).

Approximately 73% of pharmacists felt comfortable in dispensing parasiticides, while only 9.1% felt comfortable in dispensing medicines for the reproductive system. Besides, the therapeutic class for which the pharmacist did not feel comfortable at all was medicines for the cardiovascular system (21.2%) (Table 3).

The most common reason for seeking advice from pharmacists was diarrhea (100%). Owners frequently contact the pharmacist (75%) when the animal shows symptoms of pain and in 54.6% of the cases when animals vomit. According to Table 4, 50% of pharmacists were rarely consulted for asthenia (generalized weakness), and 64.7% rarely for inappetence (loss of appetite). Often, owners asked for advice from pharmacists about dosage and use of medicine (87.5% and 93.6%, respectively). On the contrary, medicine-medicine interaction was never a reason for seeking advice (54.5%) (Table 4).

Veterinary medicines dispensed in retail pharmacies were 97% for ruminants, 63.6% for poultry, 45.5% for equine, and 42.5% for companion animals. When a medicine is unavailable in the pharmacy, 66.7% of pharmacists preferred to order the medicine instead of referring the owner to another colleague (27.3%) or to the veterinarian (33.3%). Only 24.2% of pharmacists gave alternative medicines. When a medicine for human use is prescribed for an animal, 84.4% of the pharmacists had no difficulty in providing advice to the owners of the animal about the use and/or precautions to be taken.

We have found that 67.7% of pharmacists used some sources to improve their knowledge of veterinary pharmacy: 22.7% used leaflets, 19.7% used magazines or journals, and 18.2% used books.

Interestingly, 48.5% of pharmacists considered that veterinary medicines did not have any economic interest for their pharmacies. The veterinary medicine represented less than 10% of the total turnover for almost 60% of pharmacists and between 25% and 50% for only 3.1% of all respondents.

Data analyses showed that 58.1% of pharmacists had interprofessional relationship with the private veterinarian. Thirty-nine percent of these pharmacists collaborated with the private veterinarian, while 18.2% of pharmacists considered them as competitors.

Over 110 of pharmacists who were not involved in veterinary medicine dispensing (Group 2), 62.7% of them did not have courses in veterinary pharmacy during their undergraduate studies. Besides, 87% did not have training in veterinary pharmacy during postgraduate studies. Pharmacists who expressed their need to be trained were 61.1%.

Besides, 61% of the pharmacists did not dispense veterinary medicines due to limited request. In addition, 21.8% of Group 2 did not dispense these medicines due to lack of training and 5.5% due to uninteresting profit margin.

## 4. Discussion

Our study is the first research conducted in Morocco and Arabic countries, seeking to assess knowledge, attitudes, and practices of retail pharmacists regarding veterinary medicines.

### 4.1. Community Pharmacy Practice regarding Veterinary Medicine

The analysis of our results revealed that one-quarter of pharmacists dispensed veterinary medicines mainly due to limited request as veterinarians also legally dispense veterinary medicines [3]. Higher percentages of pharmacists dispensing veterinary medicines were found in Kenya (82%) [9]. In Zimbabwe, 75% of pharmacists stocked veterinary medicines and 38% dispensed veterinary medicines during six months before the survey. This was explained by the fact that Zimbabwean laws allow dispensing veterinary medicines only by pharmacists. Matema et al. showed that stocking these medicines was associated with feeling competent in veterinary pharmacy [10]. In the United Kingdom, 64% of pharmacists dispensed veterinary medicines and 55% stocked veterinary medicines in 2012. According to O'Driscoll et al., pharmacists had neither training/sufficient training in veterinary pharmacy during their undergraduate nor postgraduate studies which explained the lack of knowledge that precluded them to dispensing veterinary medicines [11].

Lower percentages of pharmacists dispensing veterinary medicines were recorded in Tanzania (15%), in Ghana (7.2%), and in Nigeria (2.2%) [12–14]. These low frequencies of dispensing medicines by pharmacists were mainly due to a lack of knowledge of veterinary pharmacy and low or limited demand [12, 13]. In Nigeria, animal owners do not get veterinary medicines from pharmacies and do not get advised by community pharmacists regarding veterinary medicine-related issues [14]. In New Zealand, McDowell et al. reported that veterinary prescriptions were dispensed in pharmacies due to the medicine's lower price compared to the price in veterinary clinics and due to the availability of a wider range of veterinary medicines in pharmacies compared to those in veterinary clinics [15].

Our results revealed that 97% of Moroccan pharmacists dispensed veterinary medicines for ruminants, and only 42.4% for companion animals. Agriculture is an important sector in Morocco, with livestock of approximately 29 million ruminants [16]. This fact explained the high amount of veterinary medicines prescribed to these animals. In Ghana, 50% of medicines sold were for canines, 35.7% for poultry, and only 7.1% for cattle/cats [13], while in the United Kingdom, 47% of pharmacists dispensed veterinary prescriptions for companion animals and only 2.15% for food-producing animals in 2004 [17]. Companion animals were also the main patients in pharmacies, as reported by O'Driscoll et al. in the United Kingdom (48%), followed by cattle (27%) and equine (20%) in 2015 [11].

At the time of the survey, parasiticides were the most sold medicines in Moroccan retail pharmacies. In Zimbabwe and in Kenya, parasiticides and antibiotics were the most sold veterinary medicines in pharmacies [9, 10], while in Finland, during a survey conducted on prescriptions of antibiotics dispensed by university pharmacies, Hölsö et al. reported that antibiotics were the most prescribed medicines for dogs and cats (53%) [18].

Some human use medicines can be dispensed to animals [11, 13, 17, 19, 20]. It was found that most pharmacists in the United Kingdom were conscious of the risk of toxicity of some human medicines used on animals when they were asked if they would dispense them for some veterinary pathologies. However, only 12% refused to deliver them [17]. In their clinical review, Frankel et al. underscored some differences between dispensing human use medicines prescribed for humans and human use medicines prescribed for animals. They recommended more vigilance in five most common situations (hypothyroidism, using prednisolone, over-the-counter medication, diabetes, and administering medication) to avoid harmful errors in dispensing human use medicines to companion animals [21]. In Finland, human medicines were prescribed for dogs and cats via university pharmacies in 31% of cases [18].

### 4.2. Retail Pharmacists' Knowledge and Attitudes towards Veterinary Medicine

In general, our study revealed that less than half of the pharmacists were highly satisfied regarding their knowledge of veterinary pharmacy. A lower percentage was found in Zimbabwe, where Matema et al. have demonstrated that 83% of pharmacists rated their knowledge as insufficient [10].

Concerning feeling comfortable, 51.5% of Moroccan pharmacists were at ease while dispensing prescribed veterinary medicines in spite of not having courses in veterinary pharmacy as undergraduate courses (69%) or training (59.4%) comes mostly from the fact that 67.7% of pharmacists got information on veterinary medicines from different sources (leaflets, magazines or journals, and books). Only 34% of pharmacists felt able to dispense in Zimbabwe [10], and 6% felt competent to give advice on animal health issues in the United Kingdom [17]. Likewise, more than 20% of pharmacists in Nigeria were not convinced that their knowledge would be suitable to dispense veterinary medicines to animals safely. Frankel et al. confirmed in their clinical review that most of Canadian pharmacists had not received suitable training to dispense veterinary prescriptions [21].

Almost 94% of Moroccan pharmacists expressed their need to improve knowledge of veterinary pharmacy. This result was in line with what Monie et al. found in their study (86%) [17]. Ogaji et al. have demonstrated that 63.3% of pharmacists in Nigeria needed more information on veterinary pharmacy. All pharmacists confirmed that further information might encourage them to get more involved in their dispensing knowledge and experience of dispensing veterinary medicines. Nevertheless, 93% of pharmacists confirmed that participating in animal dispensing service could improve their practice even though they were not considering dispensing these medicines [14]. On the contrary, 96% of pharmacists in the United Kingdom supported giving tuition in veterinary pharmacy to undergraduate student pharmacists [11].

When they were faced with dispensing veterinary medicines inquiries, 42.9% of pharmacists in Ghana preferred to refer an animal owner to a veterinary clinic, 28.6% to another pharmacist, and 21.4% to the veterinary surgeon [13]. A big number of pharmacists who referred owners to a veterinarian for additional advice were recorded in the United Kingdom (67%) [11], in Zimbabwe (69% referred patients to a veterinary surgeon or did not dispense veterinary medicines) [10], and in Tanzania (90%) [12]. Matema et al. study showed that 40% of pharmacists were advised with sufficient information and suitable tools from a veterinary surgeon when they faced problems in medicine-dispensing issues in Zimbabwe [10].

According to Justin-Temu et al., when a veterinary medicine is unavailable in the pharmacy, 59% referred the animal owner to a veterinary surgeon, 28% substituted it with another medicine, and 13% did nothing [12]. Our study showed a similar percentage: 66.7% referred them to the veterinary surgeon, 27.3% referred them to another pharmacist, and 24.2% gave an alternative medicine. A lower percentage was found in Ghana: 28.6% of pharmacists referred the animal owner to a veterinarian and 28.4% to another pharmacist [13]. In the United Kingdom, 8% of pharmacists dispensed non-prescription-only veterinary medicines in 2004 [17], and 64% sold human medicines for animals in 2012 [11]. Manifestly, the results obtained from the above studies showed that retail pharmacists preferred to refer their inquiries about veterinary medicines to the veterinary surgeon or to a veterinary clinic.

### 4.3. More Veterinary Pharmacy Training for Retail Pharmacists

Lust et al. demonstrated that an elective course for pharmacy students [22] and a postgraduate course in veterinary pharmacy for practicing pharmacists [23] were beneficial. Indeed, these courses increased pharmacists' confidence. This study was in line with the studies of Luiz Adrian et al. [24], Ngwuluka et al. [25], and Justin-Temu et al. [12]. Indeed, Justin-Temu et al. found that 79% of pharmacists reported substantial satisfaction/progress in their veterinary knowledge after having training in veterinary pharmacy [12]. Besides, Young et al. underscored the same growing knowledge of veterinary pharmacotherapy of the retail pharmacists, based on a pretest and posttest after having an educational program [26]. Our study revealed that two-thirds of pharmacists did not have courses in veterinary pharmacy during their undergraduate studies. This percentage comes from the fact that universities of pharmacy did not include veterinary pharmacy. For example, in Morocco, a ministerial decree n°2175-18 [27] has reformed the curriculum for the undergraduate pharmacy degree. Veterinary pharmacy was then introduced in the Moroccan Faculties of Medicine and Pharmacy since 2015. From the foregoing studies, it appears that having more training in veterinary pharmacy gave an appreciated impact on pharmacists' attitude and knowledge in general towards veterinary medicine and enhanced the ability to dispense the veterinary medicines as well as the human use medicines.

As has been highlighted previously in the above studies, lack of knowledge was the most relevant fact that limited the pharmacist in dispensing veterinary medicines. To fill this gap, pharmacists had to draw on knowledge from different sources of information. We have found that most of the Moroccan pharmacists (22.7%) used leaflets to check their knowledge. In comparison with the United Kingdom, the most consulted source of information was textbooks (or colleagues) (16%). Fifteen percent used the Internet, and fourteen percent consulted a veterinarian [11]. On the contrary, 36.3% of Kenyan pharmacists consulted the veterinarian or used leaflets to get informed on veterinary medicines [9]. In Tanzania, pharmacists also used different sources of veterinary medicine information such as books, Internet, conference/workshops, short training courses, and consulting a veterinarian or their colleagues [12].

This study has some limitations. First, it did not include pharmacies all over the 12 regions of Morocco. Some regions have more agriculture than other regions and therefore more veterinary prescribed medicines. Considering these findings, we believe that more attention could have been focused to assess community pharmacy practice in the remaining regions, especially in rural areas. Thus, it turns out that a memory bias may have influenced the study. The respondents were unable to remember whether they had tuition in veterinary pharmacy during their undergraduate studies. Besides, social desirability bias is more likely to emerge in qualitative questions. Social desirability bias is the tendency to respond in a pleasant way and to give socially acceptable answers [28]. Qualitative questions in our study were used to assess the experience, knowledge, and perceptions of respondents about dispensing veterinary medicines in retail pharmacies. We believe that this bias has influenced some pharmacists when answering questions about feeling comfortable in dispensing veterinary medicines.

## 5. Conclusions

Involvement of Moroccan pharmacists in dispensing veterinary medicines was limited. Forty-eight percent of the interviewed pharmacists were confident in dispensing veterinary medicines.

Despite their confidence in their ability to dispense veterinary medicines, more than 93.5% of pharmacists displayed their need to improve their knowledge of veterinary medicines.

This study revealed also that 61% of retail pharmacists did not dispense veterinary medicines due to a lack of request. It is evident from our finding and those from other countries that more veterinary training is needed among retail pharmacists.

Further surveys must be carried out in the future on a larger group of pharmacists and veterinarians, to determine the views of these professionals on the management and use of veterinary medicines. More details are needed on the practice of pharmacy to lead to changes in the policy for a better use and management of veterinary medicines. Interprofessional collaboration between the veterinarian and the pharmacist is required to provide appropriate care to animals.

## Figures and Tables

**Table 1:** Characteristics of the interviewed Moroccan retail pharmacists (*n* = 143).

Data	*N* (%)

Age (years)^*∗*^	45.8 ± 8.5
Female	53 (37.1)
Years of practicing community pharmacy^*∗*^	17.1 ± 8.2

Location of pharmacies	
(i) Urban	119 (83.2)
(ii) Rural	18 (12.6)
(iii) Suburban	5 (3.5)

Regions where pharmacies are located	
(i) Rabat-Salé-Kénitra	45 (31.5)
(ii) Casablanca-Settat	28 (19.6)
(iii) Souss-Massa	23 (16.1)
(iv) Eastern region	15 (10.5)
(v) Others	39 (42)

Faculty of pharmacy degree (country)	
(i) Eastern Europe	67 (46.9)
(ii) France	27 (18.9)
(iii) Morocco	22 (15.4)
(iv) Others	24 (16.8)

^*∗*^Expressed as mean ± standard deviation.

**Table 2:** Forms of veterinary medicines dispensing in Moroccan pharmacies (*n* = 33).

Veterinary medicines dispensed through	Very frequent, *n* (%)	Frequent, *n* (%)	Rare, *n* (%)	Never, *n* (%)	Total, *n* (%)

Veterinary prescription	0 (0)	1 (3.6)	16 (57.1)	11 (39.3)	28 (84.8)
Dispensary advice	13 (39.4)	14 (42.4)	5 (15.2)	0 (0)	32 (97)
Request of the animal owner	9 (27.3)	20 (60.6)	1 (3)	0 (0)	30 (91)

**Table 3:** Evaluating dispensing advice of veterinary medicines according to the veterinary anatomical therapeutic chemical classification system (ATC vet) (*n* = 33).

ATC vet groups	Comfortable, *n* (%)	Less comfortable, *n* (%)	Uncomfortable, *n* (%)	Total, *n* (%)

Antiparasitic products, insecticides, and repellents	24 (75)	8 (25)	0 (0)	32 (96.9)
Alimentary tract	17 (51.5)	15 (45.5)	0 (0)	32 (96.9)
Respiratory system	10 (30.3)	10 (30.3)	4 (12.1)	24 (72.7)
Urinary system and sex hormones	9 (27.3)	12 (36.4)	3 (9.1)	24 (72.8)
Dermatologicals	7 (21.2)	14 (42.4)	1 (3)	22 (66.6)
Others^*∗*^	7 (21.2)	5 (15.1)	1 (3)	13 (39.3)
Musculoskeletal system	6 (18.2)	7 (21.3)	5 (15.2)	18 (54.7)
Sensory organs	4 (12.1)	10 (30.3)	2 (6.1)	16 (48.5)
Genitourinary system and sex hormones	3 (9.1)	12 (36.3)	4 (12.1)	19 (58.5)
Metabolism	3 (9.1)	14 (42.4)	2 (6.1)	19 (58.5)
Cardiovascular system	0 (0)	8 (24.3)	7 (21.2)	15 (45.5)

^*∗*^Nontherapeutic agents.

**Table 4:** Frequency of giving advice related to symptoms and to veterinary medicines' uses (*n* = 33).

Dispensary advice	Frequent, *n* (%)	Rare, *n* (%)	Never, *n* (%)	Total, *n* (%)

Related to symptoms				
Diarrhea	31 (100)	0 (0)	0 (0)	31 (93.9)
Pain	18 (75)	4 (16.7)	2 (8.3)	24 (72.7)
Vomiting	12 (54.6)	8 (36.4)	2 (9.1)	22 (66.7)
Fever	9 (40.9)	9 (40.9)	4 (18.2)	22 (66.7)
Inappetence	3 (17.7)	11 (64.7)	3 (17.6)	17 (51.5)
Asthenia	5 (31.3)	8 (50)	3 (18.8)	16 (48.5)

Related to veterinary medicine uses				
Dosage	29 (93.6)	2 (6.5)	0 (0)	31 (93.9)
Use	28 (87.5)	4 (12.5)	0 (0)	32 (97)
Overdose	3 (13.6)	14 (63.6)	5 (22.7)	22 (66.7)
Side effects	5 (21.7)	13 (56.5)	5 (21.7)	23 (69.7)
Medicine-medicine interaction	1 (4.5)	9 (40.9)	12 (54.5)	22 (66.7)

## Data Availability

The data used to support the findings of this study are included within the article.
